# The Influence of Physical Mixing and Impregnation on the Physicochemical Properties of Pine Wood Activated Carbon Produced by One-Step ZnCl_2_ Activation

**DOI:** 10.3390/mi14030572

**Published:** 2023-02-28

**Authors:** Josphat Phiri, Hamidreza Ahadian, Maria Sandberg, Karin Granström, Thad Maloney

**Affiliations:** 1 School of Chemical Engineering, Department of Bioproducts and Biosystems, Aalto University, P.O. Box 16300, 00076 Aalto, Finland; 2 Department of Engineering and Chemical Sciences, Karlstad University, 651 88 Karlstad, Sweden

**Keywords:** pine wood, supercapacitor, activated carbon, biomass, energy storage, carbonization, physical mixing and impregnation

## Abstract

In this study, two different sample preparation methods to synthesize activated carbon from pine wood were compared. The pine wood activated carbon was prepared by mixing ZnCl_2_ by physical mixing, i.e., “dry mixing” and impregnation, i.e., “wet mixing” before high temperature carbonization. The influence of these methods on the physicochemical properties of activated carbons was examined. The activated carbon was analyzed using nitrogen sorption (surface area, pore volume and pore size distribution), XPS, density, Raman spectroscopy, and electrochemistry. Physical mixing led to a slightly higher density carbon (1.83 g/cm^3^) than wet impregnation (1.78 g/cm^3^). Raman spectroscopy analysis also showed that impregnation led to activated carbon with a much higher degree of defects than physical mixing, i.e., *I*_D_/*I*_G_ = 0.86 and 0.89, respectively. The wet impregnated samples also had better overall textural properties. For example, for samples activated with 1:1 ratio, the total pore volume was 0.664 vs. 0.637 cm^3^/g and the surface area was 1191 vs. 1263 m^2^/g for dry and wet mixed samples, respectively. In the electrochemical application, specifically in supercapacitors, impregnated samples showed a much better capacitance at low current densities, i.e., 247 vs. 146 F/g at the current density of 0.1 A/g. However, the physically mixed samples were more stable after 5000 cycles: 97.8% versus 94.4% capacitance retention for the wet impregnated samples.

## 1. Introduction

Due to the increase in energy costs, depletion of fossil fuels and climate change caused by excessive greenhouse emissions, the need for renewable and sustainable energy sources has never been so critical. It is therefore not surprising that the production of green and clean energy from biomass and biofuels, solar, wind, and hydro power has exponentially increased in recent years [1]. To fully benefit from the generated green and clean energy from these sources, an efficient and renewable energy storage system is also required.

The two most common electrical energy storage systems are batteries and supercapacitors. Electrodes are one of the most important and common components in these devices. The electrode plays a major role in the performance and type of energy storage system. Carbon-based materials, especially those derived from activated carbon (AC), are predominantly used as electrode materials due to their relatively low cost, high surface area, and excellent chemical and thermal stability [2,3]. Apart from energy storage, activated carbon, due to its easily tunable properties, can also be used in other applications such as gas adoption, air purification, water treatment and salination [4,5,6,7].

It has been well-established by many studies that the precursor material and activation process play a major role in determining the physicochemical properties of the activated carbon [8,9]. In recent years, there has been a strong movement towards the production of activated carbon from materials with low environmental impact, such as biomass [10,11,12]. In fact, there are many studies reporting biomass-derived activated carbon from nanocellulose, peanut shells, coffee beans, willow, cassava peel etc. with a range of properties for various applications [13,14,15,16,17,18,19,20]. The main attraction of using biomass as a carbon source is low cost, abundance, renewability and sustainability etc. [21].

One of the most important processes during biomass carbonization is activation. The activation process involves a reaction between the precursor and the activation agent to produce a porous structure. This reaction can take place during the impregnation stage and during the heat treatment. The properties of the produced activated carbon can be modified by different activation processes. Activated carbon can be prepared by different activating agents such as KOH, ZnCl_2_, NaOH, H_3_PO_4_, HNO_3_, CO_2_ etc. [21,22].

The strong acids or bases and the products of reactions can be corrosive to the reactors which increases the cost of the synthesis process. In recent years, efforts have been made to find highly efficient chemical activators. Although these chemical activators cannot be considered green reagents, they offer an opportunity to develop new activation strategies for the synthesis of porous carbons. These novel activators produce porous carbons with high specific surface area and unique morphology but generate toxic gases that require further purification. These novel chemical reagents can be classified into three categories according to their different activation mechanisms [23]: molten salts (CuCl_2_, NiCl_2_, NaCl, KCl, and FeCl_3_), decomposable salts (zinc acetate, Zn(NO_3_)_2_, NaNO_3_, calcium acetate, sodium acetate, MgCO_3_ etc.)., and oxidized salts (HNO_3_, KMnO_4_, KNO_3_, Mn(NO_3_)_2_ etc.) [24,25,26,27,28,29,30,31]. During the molten salt carbonization and activation process, molten salts can act as a catalyst to speed up the conversion of biomass to biocarbon, can serve as a physical barrier to stop direct contact and consequent reactions between carbon and the atmosphere, and function as a fluid carrier for mass transfer [32]. The reader is referred to [22,23,32] for more comprehensive reviews of different activation agents including salts.

In this study however, the traditional ZnCl_2_ was chosen as the activation agent. Zinc chloride is widely used to produce activated carbon, especially from lignocellulosic precursors. One of the widely used chemical reagents for the synthesis of porous carbon with tunable pore structure is KOH. KOH activated carbons have generally high pore volume and surface area. However, due to high costs, corrosion of reactors, poor carbon yields, severe contamination etc., utilization of KOH is not universally suitable [33]. As an alternative to KOH, ZnCl_2_ is neutral, less corrosive, low cost and does not require high activation temperature [34,35].

One variable that can affect the properties of activated carbon, which has not been investigated in detail, is the physical mixing or impregnation of the precursor with zinc chloride. “Physical mixing” means mixing the precursor with an activation agent in the absence of water, i.e., dry activation powder mixed with the dry pine wood powder, whilst “impregnation” implies first dissolving the activator in water followed by mixing the solution with the precursor.

Pine wood was chosen as a biomass precursor due to low cost and availability in large quantities as waste products from forest and agriculture industries. The pine wood pellets received from Laxå Pellets are manufactured from residual wood products from sawmills and contain only debarked pine wood. These pellets are normally used as heating pellets for heat production.

This paper compares the effects of physical mixing and impregnation of ZnCl_2_ pine wood activated carbon on various functional properties. The physical and chemical properties of the produced activated carbon are explored by XPS, SEM, Raman spectroscopy, pycnometer, etc. The activated carbons are also compared as electrode materials in supercapacitor application.

## 2. Experimental

### 2.1. Materials and Preparation

Zinc chloride, poly(vinylidene fluoride) (PVDF), N-methyl-2-pyrrolidone (NMP) were purchased from Sigma Aldrich. Carbon black Super P was purchased from Nanografi. The pure pine sawdust material was obtained from the Swedish company Laxå Pellets.

A one-step carbonization and activation method was used to prepare the activated carbon from pine wood. Pine wood powder was directly mixed with ZnCl_2_ in wet and dry form in the mass ratio of 1:1 and 1:2. Samples produced by impregnation are designated as WET-1 and WET-2, the numbers in front correspond to the mass ratio of pine wood to ZnCl_2_. In this process, ZnCl_2_ was first dissolved in water and the pine wood powder was soaked in the solution for 4 h and subsequently dried in an oven at 105 °C until completely dry. In the physical mixing procedure, the dry powders of pine wood and ZnCl_2_ in predetermined quantities were thoroughly mixed in the ration of 1:1 and 1:2 before the carbonization process. These samples are designated as DRY-1 and DRY-2, the numbers in front represent the ratio of pine wood to ZnCl_2_. As a reference, pine wood was also carbonized without the addition of ZnCl_2_. This sample is referred to as Ref-600C.

The samples were then carbonized by increasing to 600 °C at a heating rate of 5 °C/min in a nitrogen atmosphere followed by 2 h isothermal treatment at this temperature. The carbonized samples were then washed with 0.5 M HCl and excess deionized water until neutral pH. The washed samples were then dried in an oven at 105 °C before further analyses.

### 2.2. Materials Characterization

Scanning electron microscopy was used to analyze the surface structure and morphology of the prepared activated carbon powders using a Zeiss Sigma VP. The samples were sputtered with gold-palladium film prior to the SEM measurements. Raman spectra were collected using a Renishaw inVia™ Qontor Confocal Raman Microscope equipped with a 532 nm excitation laser. The density of the samples was determined by helium pycnometer using a Quantachrome Ultrapyc 1200 e. Surface area and pore volume were determined using a Micromeritics Tristar II 3020 apparatus based on the gas sorption method. X-ray photoelectron spectroscopy (XPS) was conducted using a Kratos Axis Ultra ESCA system with a monochromatic Al-Kα source. The thermal stability of the carbon powders was characterized by thermogravimetric analysis (TA Instruments Q500). The measurements were conducted under a nitrogen flow rate of 60 cm^3^/min over a temperature range of 30–900 °C at a heating rate of 10 °C/min.

The electrochemical properties were studied using a conventional three-electrode systems. The active carbon material, Pt wire and Ag/AgCl electrode represents the working, counter, and reference electrodes, respectively. The slurry for the working electrodes was prepared by mixing 80 wt% as prepared carbon materials with 10 wt% conductive carbon black Super P and 10 wt% PVDF dissolved in NMP. The prepared slurry was then coated on nickel foam, dried at 105 °C for at least 48 h and pressed before the electrochemical measurements in 6 M KOH aqueous electrolyte. The active electrode was prepared with a high mass loading of about 10 mg/cm^2^ as recommended [36,37]. All the electrochemical tests were carried out at room temperature. Galvanostatic charge/discharge, cycling tests, cyclic voltammetry (CV), and electrochemical impedance spectroscopy (EIS) measurements were carried out in a Gamry Reference 600+ potentiostat/galvanostat/ZRA.

## 3. Results and Discussion

**Figure 1:**
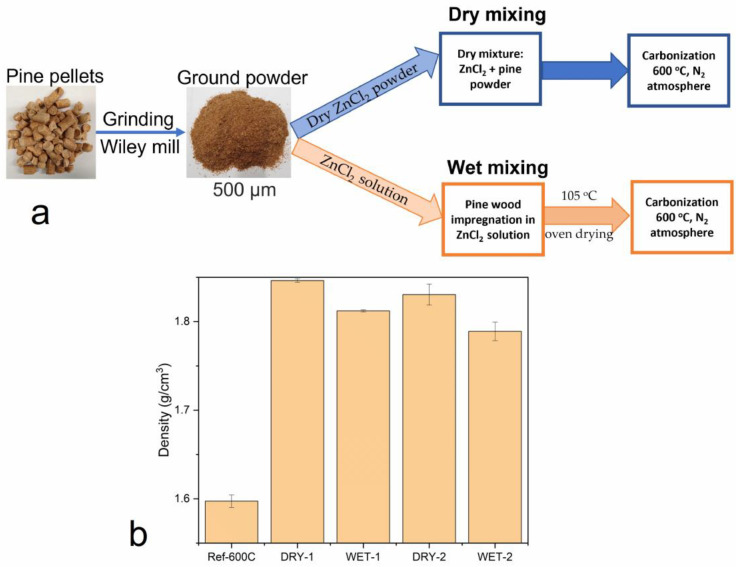
(**a**) Schematic diagram showing the two methods synthesis of activated carbon and (**b**) comparison of the density of the activated carbon from wet and dry activation methods.

In this work, zinc chloride was utilized as an activation agent for the lignocellulosic precursor as shown in Figure 1a. During the activation process, zinc chloride acts as a dehydration agent and does not prevent the release of volatiles via ZnCl_2_-saturated pores from the carbon surface [22]. It has been shown also that ZnCl_2_ causes cellulose to swell and increases the surface area after activation [38,39]. In addition, ZnCl_2_, creates interspaces between the carbon layers, which can lead to higher microporosity [40].

The density commonly referred to as “helium density” of the carbons obtained from dry and wet activation was compared as shown in Figure 1. Compared to the inactivated sample, there was a significant increase in density for both dry and wet activated carbons. The activation ratio, however, had a very small effect on the density. At high activation ratio, there is enough activator to react with and etch away the surface of the precursor; thus, a slight decrease in density is observed. At both ratios, the density of the samples activated with the physical mixing was a bit higher than the density of the impregnated samples.

The relative increase in the density of the activated carbons compared to the inactivated carbon is an indication of removal of lighter volatile materials from the carbon structure. During this process, the pore structure is opened, and the ash content increases, leading to densification of the carbon matrix. It has also been suggested that an increase in density could indicate the ordering of the layered planes caused by the increased aromaticity [41]. The wet activated carbons showed a slightly lower density than the dry activated samples because the impregnation method is more efficient in reacting with the precursor, i.e., the activating solution can penetrate inside the small pores of the raw material, etching away the surface and thus, creating more volume. The lower density might also indicate the presence of hollow particles in impregnated samples, i.e., a solid shell with empty spaces inside the particles [42,43].

**Figure 2:**
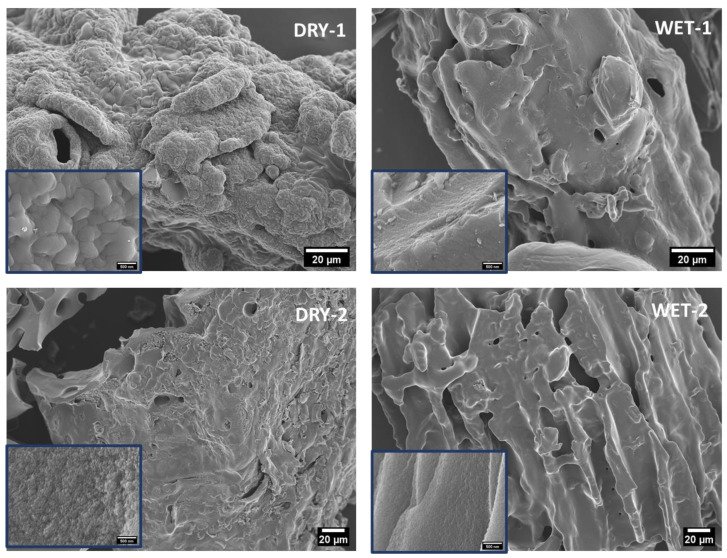
SEM images of the prepared samples with the inserts showing a magnified central area. (Scale bar for the inserts is 500 nm).

Figure 2 depicts the SEM images of the produced activated carbon. The carbon samples present a visibly porous microstructure with cracks and holes, which might suggest the presence of a large pore volume and high specific surface area. During carbonization, most of the organic volatiles were removed leaving behind a structure with various pores and cavities. A more pronounced porous surface can be observed at high magnification (Figure 2). Especially in electrochemical applications, activated carbons with a porous structure is essential because it may generate a high specific surface area, provide abundant adsorption areas for the ions which, promotes rapid ion diffusion within the electrode and between the electrode and electrolyte, thus enhancing the capacitive performance. The surface of the dry samples shows a more porous structure than the wet samples. This is because the ZnCl_2_ solution can penetrate inside the pine wood, i.e., evenly distributed on the biomass. However, for the dry ZnCl_2_ powder, it is first in contact with the surface of biomass, i.e., high activation effect or etching on the surface before the molten salt at high temperature can penetrate inside the biomass. The activation process of dry ZnCl_2_ powder and dry biomass begins on the surface. When the salt becomes molten at high temperature it penetrates inside the precursor.

**Figure 3:**
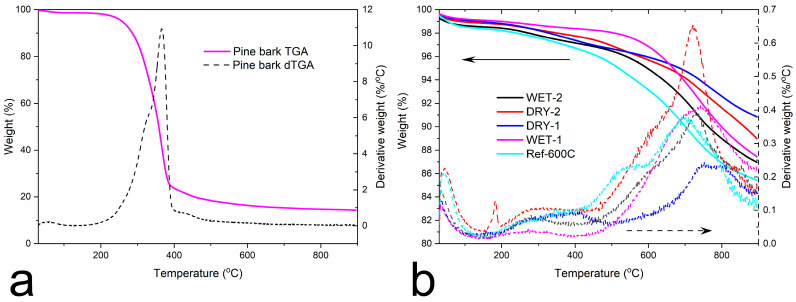
Thermal gravimetric analyses in N_2_ of (**a**) pine wood and (**b**) carbonized samples.

The thermal behaviors of the pine wood and carbon samples with physical mixing and impregnation with ZnCl_2_ at ratios of 1:1 and 1:2 were investigated by TGA. The TGA and dTGA curves of pine wood and carbonized pine wood are distinctively different (Figure 3). The weight loss in the first region up to around 150 °C is due to water loss by evaporation for both set of samples. The pine wood (Figure 3a) has an additional pronounced weight loss from around 200 to 400 °C. This weight reduction is associated with the dehydration and release of volatiles from the cellulosic structure as well as the decomposition of lignin, cellulose and hemicellulose molecules [44,45].

For the carbonized samples, the degradation process can be divided into three stages. The first stage is the initial weight loss up to about 200 °C caused by the evaporation of adsorbed water. The second stage from about 200–550 °C is attributed to the slow thermal decomposition of the organic carbon structure [46]. The third stage of degradation from 600–900 °C is believed to be caused by secondary degassing, manifested on the dTGA with maximum at around 750 °C. The lowest yield was recorded for the reference sample without the activation agent. The activation process also enhances the removal of volatiles; thus, higher yields were achieved. The dry and wet impregnation process had little effect on the final yields. All the activated samples showed a similar degradation profile and final weight loss count.

**Figure 4:**
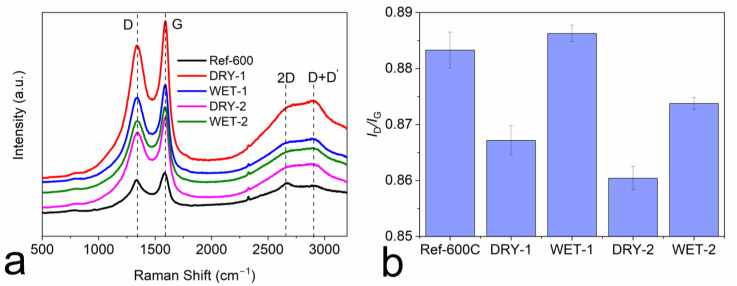
(**a**) Raman spectra of the wet and activated carbon samples and, (**b**) The intensity ratio of G band to D band (*I*_G_/*I*_D_).

Raman spectroscopy was utilized to analyze the crystal structure and defects of the produced carbons [47], as shown in Figure 4. All the produced carbons show more pronounced G- and D-bands at around 1327 and 1585 cm^−1^, respectively, and the severely diminished 2D and D + D′ bands at around 2655 and 2885 cm^−1^, respectively (Figure 4a). The G-band corresponds to the vibration of sp2 hybridized carbon atoms in a hexagonal lattice. The D-band is due to the presence of defects and disorders in the carbon structure [48]. The positions of the G and D band were constant in both wet and dry activated carbons. The ratio of the precursor to the activator did not have any effect on the positioning of the bands. The intensity ratio of the G and D band (*I*_D_/*I*_G_) can be used to quantify the number of defects in the carbon structure. It is clearly seen from Figure 4b that there was a dependance of defect quantity if the system was activated with dry or wet activator as well as the activation ratio. The wet impregnated samples show a bit higher quantity of structural defects than the dry counterpart. The etching mechanism of the wet activator on the surface of the precursor is more severe than the dry activator. The whole surface of the precursor is wetted by the activation solution, which reacts and causes etching and development of nanopores on the surface, which could be detected as defects by Raman spectroscopy. The same process occurs with the dry activator. However, it is not as homogeneous as in the wet form and does not affect the precursor surface as much as using the wet activating solution. Thus, the number of defects is lower. It is also important to note that the edges contribute to the defects. Therefore, the surface with more pores has more boundaries, which can be detected by Raman spectroscopy as defects [49,50].

**Figure 5:**
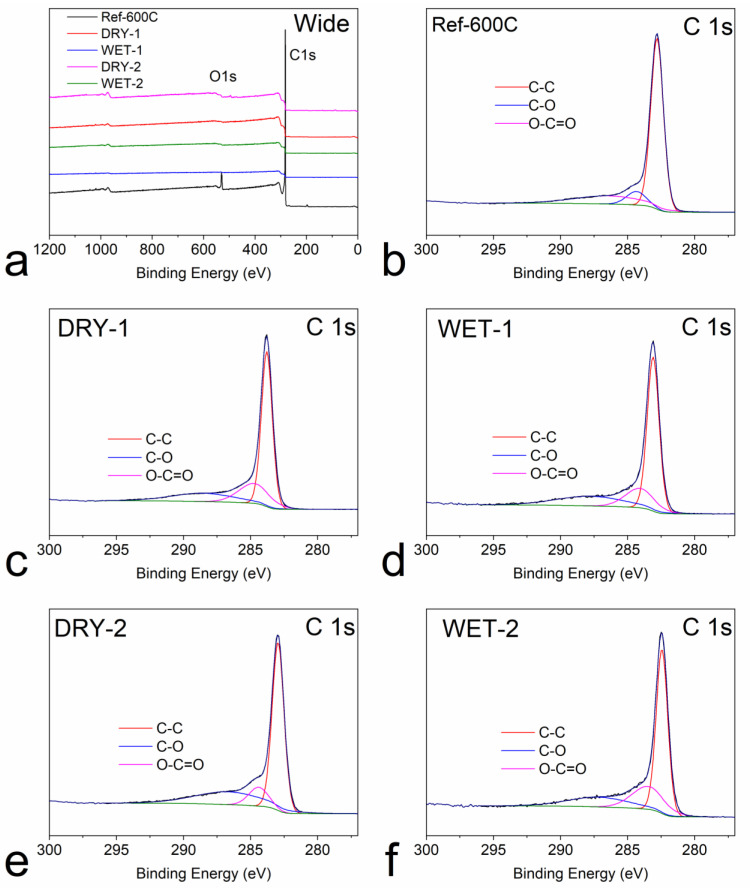
XPS spectra (**a**) wide spectra for all samples and C 1 s for (**b**) Ref-600C (**c**) DRY-1 (**d**) WET-1 (**e**) DRY-1 and (**f**) WET-2.

The chemical composition of the carbonized samples was analyzed by XPS. The wide survey XPS spectra in Figure 5a for all samples exhibit two distinct peaks at around 285 eV and 532 eV belonging to the C1s and O1s, respectively. The surface parameters of the samples are summarized in Table 1. A high amount of carbon was present in all the samples. Dry mixing and impregnation had very little effect on the amount of oxygen or carbon. However, a slight increase of carbon content is observed for the wet impregnated samples. This might indicate that the impregnation method is more efficient in removing the volatiles than the physical mixing. The activation ratio also had a slight effect on the amount of oxygen. When activation ratio increased from 1:1 to 1:2, the oxygen content for the dry and wet increased by 5.3 and 3.2 at%, respectively. This might be due to the increased pore volume and surface area of the samples, which provides the availability of more sites for the residual oxygen. The deconvolution of the C1s spectra (Figure 5a–f) reveals the presence of three components, located, respectively, at 284 eV (C=C), 285 eV (C-O) and 289 eV (O-C=O) [51]. It can be seen also from Table 1 that all the samples to a large extent contain sp^2^ hybridized carbon atoms evidenced by the large area occupied by the C=C atoms [4,52]. The physical mixing and wet impregnation method had very little effect on the amount of these components.

**Figure 6:**
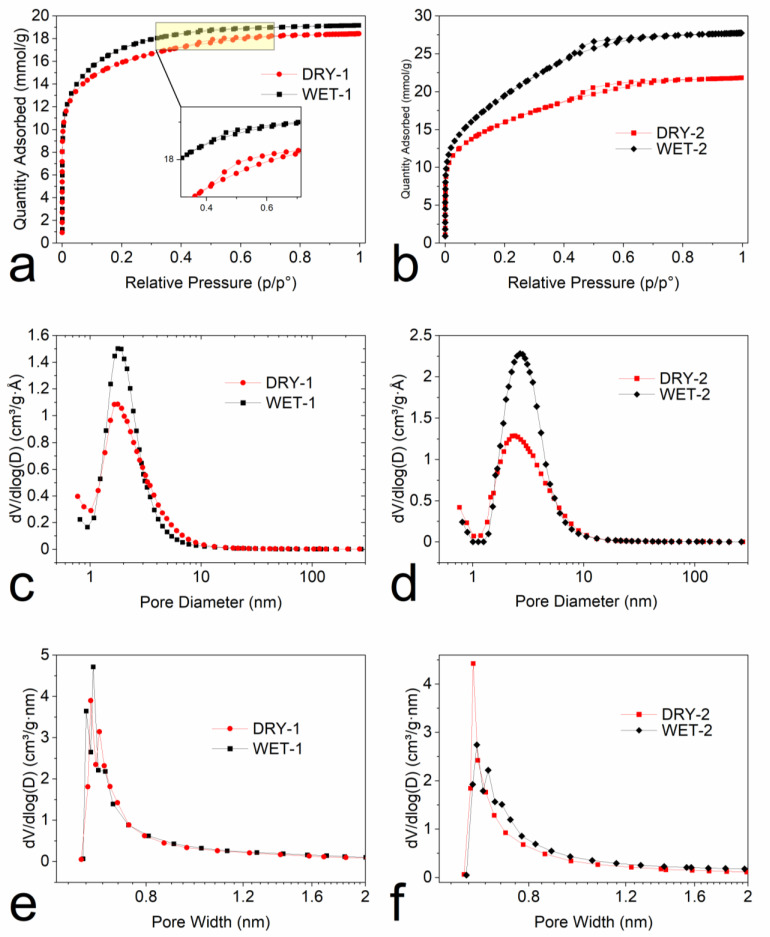
(**a**,**b**) the adsorption–desorption isotherms, (**c**,**d**) the pore size distribution (Dollimore-Heal method) and, (**e**,**f**) micropore distribution (Horvath-Kawazoe method).

The pore and surface information such as structure, volume, area, and distribution play a significant role in the performance of the activated carbon. The parameters can be easily influenced by the method of activation. The effect of wet and dry activation methods on the surface and pore parameters is investigated. Figure 6a,b shows the nitrogen adsorption–desorption isotherms at 77 K and the textural properties of the samples are shown in Table 2. It can be clearly seen that all the samples show a high amount of nitrogen adsorbed at low relative pressures which indicates the presence of micropores. Samples activated with a weight ratio of 1:1 in Figure 6a, show type I isotherms, which are basically microporous materials, but also show a small hysteresis loop (Figure 6a inset) in the adsorption–desorption isotherms also indicating the presence of mesopores. The samples activated with a weight ratio of 1:2 show a type-IV isotherm (Figure 6b), with a hysteresis loop in the in the *p*/*p*^0^ region of 0.4–0.9 indicating the presence of a large fraction of mesopores. The pore size distribution (PSD), (Figure 6c,d), shows peaking at 1.6 nm and 2.5 nm with activation ratio 1:1 and 1:2, respectively. There is no difference in the PSD between the dry and wet impregnation methods, although the wet impregnated sample had a slightly higher total pore volume (Table 2). Increasing the activation ratio to 2 leads to the broadening or widening of the pores (Figure 6d). The availability for more activating agent means more surface can be etched away. By increasing the amount of ZnCl_2_, the development of mesopores is also enhanced. The micropores deform and transform into mesopores (Table 2). This behavior was also observed in other studies with an increase in ZnCl_2_ [40,53]. In addition, there is also a slight difference in the PSD between the dry and wet impregnated samples. The peak shifted from 2.2 nm to 2.7 nm for DRY-2 and WET-2, respectively. The wet impregnation solution can penetrate inside the small pores of the precursor and as such, it reacts with the whole precursor surface leading to the development of more and bigger pores. This is also evident from the amount of mesopores, 0.45 cm^3^/g for the DRY-2 and 0.75 cm^3^/g for the WET-2. The micropore distributions according to the Horvath-Kawazoe model (Figure 6e,f), were similar.

**Figure 7:**
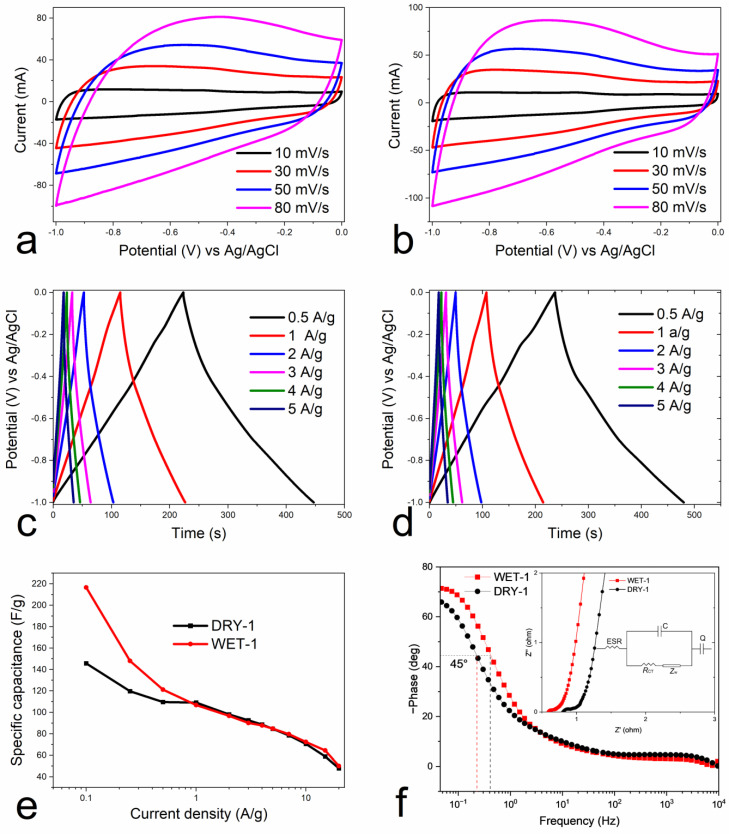
Electrochemical measurements: the CV curves of (**a**) DRY-1, (**b**) WET-1. Charge–discharge cycles of (**c**) DRY-1, (**d**) WET-1, (**e**) specific capacitance as a function of current density and (**f**) Bode plot and the inset showing the Nyquist plots with the equivalent circuit model.

The electrochemical performance of the samples was conducted in a three-electrode system using a 6 M KOH aqueous solution as the electrolyte. Cyclic voltammetry (CV), galvanostatic charge–discharge and electrochemical impedance systems (EIS) were employed for the analysis. The CV of the samples is shown Figure 7a,b. The measurements were performed at room temperature within the potential window of 0 to −1 V. The more or less quasirectangular shape of the CV curves (Figure 7a,b) at different sweep rates indicates good reversible electric double-layer capacitive behavior [54]. The WET-1 sample, however, showed a much better rectangular form, indicating better electrochemical performance than the DRY-1 sample, which is consistent with the analyzed textural properties.

The galvanostatic charge/discharge cycle (GCD) curves measured at the current densities 0.5, 1, 2, 3 4, and 5 A/g are shown in Figure 7c,d. It was found that for both dry and wet impregnated samples, the charge and discharge profiles at different densities exhibit the characteristic triangular form with rising/falling potentials at turning points, which reflects good charge and discharge capacitive behavior. The linear and symmetrical GDC curves observed, even at high current density, indicate the good electrochemical reversibility and low voltage drop at the start of the discharge curve.

Figure 7e shows the dependence of specific capacitance on current density. The gravimetric capacitance (*C_g_*) from the charge–discharge cycles for the three-electrode system was calculated using Equation (1) [55]

(1) $$C_{g}=\frac{I\times ∆t}{m\times ∆V}$$

where *I* (A) is the discharge current, Δ*t* (s) is the discharge time, Δ*V* (V) is the voltage change excluding the IR drop during the discharge process, and *m* (g) is the mass of the active material. The specific capacitance of WET-1 samples at low current density is much higher than that of the DRY-1 sample. For example, at the current density of 0.1 A/g the specific capacitance of WET-1 and DRY-1 sample is 247 vs. 146 F/g, respectively. However, as the current density increases, the specific capacitance remains the same between the two samples. This slight disparity could be due to the difference in micropore volume according to the Horvath-Kawazoe model (Table 2). At lower current density, the micropores of WET-1 can be easily accessed and thus, contribute to the higher capacitance. However, at higher current density, the accessibility to the micropores is limited. This shows that the efficiency of ionic transfer within the electrode is poor. The capacitance decreases with the increase of current density and it drops to 59 F/g at the high current density of 15 A/g for the DRY-1 sample representing 60% loss and for the WET-1 sample, 64 F/g, representing 70% loss. Dry activation of pine wood with ZnCl_2_ can create a pore structure that promotes a high rate of ion transfer inside and between the electrode and electrolyte, which, in turn, increases the electrochemical performance. This reduction in capacitance at higher current densities is common for heteroatom-doped carbon materials [56] and is believed to be caused by the so-called electrolyte starvation effect [57]. The presence of oxygen moieties on the surface of the electrode promotes the formation of oxygenated complexes that can cause a partial blockage of the pore entrances. This explains the decrease in capacitance at high current density, since the ions require more time to penetrate inside the electrode [57]. Moreover, poorly developed pore structure could also explain the reduction of capacitance at high current density. It is hypothesized that at high current density, the electrolyte ions do not have enough time to penetrate the deep pores and access all the surface area. Therefore, only the big pores are able to store the charge and thus, much lower capacitance is observed at high current densities [58]. Table 3 shows the comparison of the prepared samples with other biomass-derived activated carbons.

The electrochemical performance of the electrodes was further analyzed by EIS. Figure 7f shows the Nyquist plot in a frequency range from 0.01 Hz to 1 MHz, where the *Z*′axis displays the real part representing ohmic resistance, while *Z*″ the imaginary part represents the presence of non-resistive elements. In the low-frequency region, both WET-1 and DRY-1 samples exhibit an almost vertical-line, indicating a nearly ideal capacitive behavior and low diffusion resistance of electrolyte ions in the electrode material. The intersection with the *x*-axis in the high frequency region represents the equivalent series resistance (ESR), i.e., inherent solution resistance (*R*_s_) between the electrolyte and the electrode materials [26]. The ESR of 0.60 and 0.81 Ω for WET-1 and DRY-1 samples, respectively, are observed at the real axis intercept. The diameter of the semicircle in the high-frequency region represents the charge transfer resistance (*R*_ct_) and the 45° incline line in the high–medium frequencies, corresponds to the Warburg impedance [69]. The low ESR values, small semicircle diameter and low Warburg impedance indicate that the WET-1 sample has low internal resistance and a much better ion diffusion capability than the DRY-1 sample. The Nyquist plot was fitted with an equivalent circuit model, as presented in the inset of Figure 7f. ESR is equivalent series resistance, *R*_ct_ is charge-transfer resistance, *Z*_W_ is the Warburg resistance, which is related to the ion diffusion or transport in the electrolyte and *C* and *Q* are related to the double layer and faradaic capacitance, respectively [70,71]. The bode plot in Figure 7f shows the dependence of the phase angle on the frequency. The characteristic frequency *f*_0_ for a phase angle of −45° corresponds to the time constant (τ_0_ = 1/*f*_0_) of 0.37 s for WET-1 and 0.21 s for DRY-1 samples. This frequency represents the point at which the resistive and capacitive impedance components are equal, and, at frequencies higher than *f*_0_, the supercapacitor transitions to a more resistive behavior [72]. It seems that wet impregnation is able to promote the formation or development of a structure that supports good electrochemical performance. The complete wetting of the precursor surface with the activator during carbonization leads to the development of a homogeneous nanoporous structure on the surface, which could in turn promote a more efficient ionic exchange between the electrolyte and the electrode and also within the electrode.

**Figure 8:**
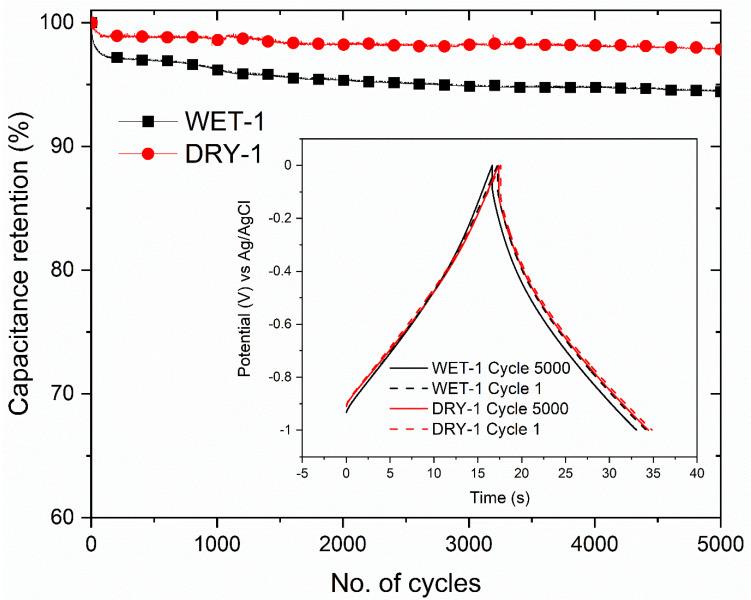
Cycling stability tests at a current density of 5 A/g with the insert showing the charge–discharge cycles at the start and end of test.

The cyclability of the electrodes measured at high current load of 5 A/g is displayed in Figure 8. The capacitance retention of the WET-1 and DRY-1 samples after 5000 cycles is 94.4% and 97.8%, respectively. Both samples show excellent capacitance retention capability although the DRY-1 sample was a bit higher. The insert shows the charge–discharge cycles at the beginning and end of cycles. It is clear that the shape is not altered for both samples, indicating excellent cycle stability. Micropores contribute the largest part of energy storage, and when they get blocked, capacitance is reduced. This might explain a slightly lower retention for the WET-1 sample in comparison to the DRY-1 sample after 5000 cycles.

## 4. Conclusions

This study compares the physicochemical properties of pine wood activated carbon produced by ZnCl_2_ activation using two methods: physical mixing and wet impregnation. Physical mixing is the waterless mixing of dry ZnCl_2_ and pine wood powders whilst wet impregnation involves dissolving ZnCl_2_ in water and mixing the solution with pine wood. Dry mixing produced denser carbons than wet impregnation, i.e., 1.83 g/cm^3^ vs. 1.78 g/cm^3^, respectively. Raman spectroscopy analysis also showed that wet impregnated carbons have a higher degree of defects than dry mixing. The wet impregnated samples also had better overall textural properties, i.e., higher pore volume (0.664 vs. 0.637 cm^3^/g) and surface area (1191 vs. 1263 m^2^/g). In terms of electrochemical application, specifically in supercapacitors, wet impregnated samples showed a much better capacitance at low current densities. For example, at the current density of 0.1 A/g, the specific capacitance is 247 and 146 F/g for wet and dry samples, respectively. However, the dry mixed samples were more stable after 5000 cycles, 97.8% versus 94.4% for the wet impregnation. Both methods offer advantages and disadvantages and, depending on the type of application, one method could be more suitable than the other. Obviously, the dry method is more preferred in terms of simplicity and low energy cost incurred from drying. However, when there are specific properties required, wet impregnation could also be used.

## Figures and Tables

**Table 1:** Surface chemical composition determined by XPS in at%.

Samples	C	O	C=C	C-O	O-C=O
Ref-600C	93.6	5.8	71.8	8.9	19.3
DRY-1	96.3	3.3	59.7	21.6	18.8
WET-1	97.0	2.7	59.9	18.6	21.9
DRY-2	90.5	8.6	61.4	11.5	27.1
WET-2	93.7	5.9	58.3	22.9	18.8

**Table 2:** Textural properties of the carbonized samples.

Samples	Ref-600C	DRY-1	WET-1	DRY-2	WET-2
BET surface, (m^2^/g)	35	1191	1263	1214	1516
t-plot *SA*_micro_, (m^2^/g)	17	551	461	437	433
t-plot *SA*_Ext_, (m^2^/g)	17	639	800	777	1083
t-plot *V*_micro_, (m^3^/g)	0.005	0.197	0.150	0.109	0.045
*V*_total_, (m^3^/g) *	0.019	0.637	0.664	0.753	0.954
BJH adsorption, cm^3^/g	0.013	0.342	0.368	0.599	0.905
BJH desorption, (cm^3^/g)	-	0.256	0.238	0.513	0.784
D-H adsorption, (cm^3^/g)	0.013	0.543	0.582	0.699	0.947
D-H desorption, (cm^3^/g)	-	0.237	0.219	0.482	0.751
Horvath-Kawazoe at 0.31, (cm^3^/g)	0.017	0.578	0.626	0.610	0.767
BJH adsorption pore width, (nm) **	2.28	2.603	2.399	2.949	2.836

* Total pore volume measured at *p*/*p*^0^ 0 = 0.995. ** Average pore diameter of porous carbon calculated by 4*V*T/*S*.

**Table 3:** Comparison of physicochemical properties of various biomass derived carbon electrodes.

Biomass	Activation Agent	Surface Area m^2^/g	Electrolye	Specific Capacitance	Capacitance Retention	Reference
Banana fibers	KOH	957	1 M KOH	324 F/g at 10 mV/s	100% at 25 A/g 5000 cycles	[59]
Coconut shell	ZnCl_2_	1874	6 M KOH	268 F/g at 1 A/g	99.5% at 3 A/g 5000 cycles	[60]
Nori	ZnCl_2_	832	6 M KOH	220 F/g at 0.1 A/g	96.6% at 2 A/g 5000 cylces	[61]
Ramie	ZnCl2	1616	6 M KOH	287 F/g at 0.05 A/g	93% at 0.1 A/g 1000 cycles	[62]
Willow wood	KOH	2793	6 M KOH	394 F/g at 1 A/g	94% at 5 A/g 5000 cycles	[63]
Rice husk ash	HF	786	6 M KOH	260 F/g at 1 A/g	84% at 20 A/g 10,000 cycles	[64]
Banana Fibers	ZnCl_2_	1097	1 M Na_2_SO_4_	74 F/g at 0.5 A/g	88% at 0.5 A/g 500 cycles	[65]
Bamboo char	KOH	3061	6 M KOH	258 F/g at 0.1 A/g	92% at 2 A/g 3000 cycles	[66]
Pine wood	-	76	2 M KOH	328 F/g at 0.2 A/g	99.7% at 4 A/g 2000 cycles	[67]
Wheat straw	KOH	2115	3 M KOH	294 F/g at 1 A/g	97.6% at 10 A/g 5000 cycles	[68]
Pine wood	ZnCl_2_ (DRY-1)	1191	6 M KOH	146 F/g at 0.1 A/g	97.8% at 5 A/g 5000 cycles	This work
Pine wood	ZnCl_2_(WET-1)	1263	6 M KOH	247 F/g at 0.1 A/g	94.4% at 5 A/g 5000 cycles	This work

## Data Availability

Data available by request from the corresponding author.
